# Modeling the impacts of natural and human factors on the hatching success of the loggerhead sea turtle *Caretta caretta* along the coasts of Italy

**DOI:** 10.1371/journal.pone.0320733

**Published:** 2025-04-09

**Authors:** Luca Ceolotto, Sandro Mazzariol, Guido Pietroluongo, Cinzia Centelleghe, Carlotta Mazzoldi, Alberto Barausse

**Affiliations:** 1 Department of Comparative Biomedicine and Food Science, University of Padua, Legnaro, Italy; 2 Interuniversity Center for Cetacean Research (CIRCE), Genova, Italy; 3 Department of Biology, University of Padova, Padua, Italy; 4 National Biodiversity Future Center (NBFC), Palermo, Italy; HUN-REN Centre for Ecological Research, HUNGARY

## Abstract

Coastal biodiversity is globally threatened by climate change and human pressures, including habitat destruction, overfishing, eutrophication, and pollution, which alter natural coastal ecosystem processes. *Caretta caretta*, hereafter referred to as loggerhead sea turtle, is listed as “Vulnerable” at global level and “Least Concern” in the Mediterranean Sea by the International Union for Conservation of Nature (IUCN). This species is the only sea turtle nesting along Italian coasts, making it crucial to understand the factors affecting its reproductive success for effective conservation strategies. However, key aspects of the ecology and life cycle in Italian waters, such as spatial distribution, reproductive site selection, and factors influencing reproductive outcomes and migratory movements, remain unknown. This study aimed to identify factors influencing the reproductive success of the loggerhead sea turtle. Data from 237 nests between 2019 and 2023 across 14 Italian regions were recovered, quality-checked, and analyzed. A statistical model predicting reproductive success, represented by hatching success, was developed, incorporating various environmental variables from marine and terrestrial spheres, along with local pressures from urbanization and anthropization. These predictors were related to hatching success using a generalized linear model (GLM) accounting for zero-inflated data. The best models identified both environmental variables, such as temperature and extreme wave events, and human-controlled factors, including the presence of dunes and coastal urbanization, as key predictors of hatching success. Coastal anthropization and beachfront disturbances were particularly important. While human activities can pose notable challenges to the loggerhead sea turtle, their identification also offers opportunities for enhancing its reproductive success through targeted management actions focused on mitigating pressures. Our findings highlight the urgent need for targeted conservation efforts to address both local and global challenges to protect and enhance the reproductive success of the loggerhead sea turtle and possibly other coastal species. Effective management can and should focus on mitigating human-induced pressures. Policymakers and conservationists need to work together to implement strategies that consider both the immediate human impacts and the long-term effects of climate change, ensuring the sustainable management of coastal ecosystems and the protection of endangered species like the loggerhead sea turtle.

## Introduction

Highly mobile marine species are key conservation targets as they frequently encounter intense human pressures during long-range movements, which can be challenging to manage since they happen across regulatory boundaries [1,2]. Additionally, some large mobile species, like tunas, are commercially valuable, while others are charismatic and protected, such as the loggerhead sea turtle which is classified globally as Vulnerable by the International Union for Conservation of Nature IUCN [3], with the Mediterranean population listed as Least Concern [4]. Long-term studies on loggerhead turtles in the Mediterranean Sea have provided valuable insights into their reproductive output, including metrics like clutch size, nesting success, and hatching rates [5]. However, similar comprehensive data are largely lacking for Italy, despite the increasing importance of its coastlines as nesting sites. This gap highlights the need for focused research to better understand reproductive patterns and inform conservation efforts in this country. During the last few years, there has been a significant increase in loggerhead sea turtle nests recorded along the Italian coastlines. This pattern could be related to the raised awareness and monitoring efforts and the increasing availability of communication tools, but it could also indicate an upward trend in the nesting activity of this species in Italy [6]. Many factors could explain the increase of the nesting activities in some areas such as a shift in the species distribution due to global warming, especially for sexually mature individuals [7]. This expansion could lead to additional threats such as unfavorable environmental conditions or new conflicts with local human activities [8] which may be particularly high along the heavily anthropized coasts of Italy [9].

Coastal zones represent a critical nexus of biodiversity and ecosystem service provision, yet they face escalating threats from anthropogenic pressures and climate change [10]. A good example is the complex reproductive behavior of sea turtles, which makes them particularly vulnerable to human pressures. Although many studies [7,11–17] have focused on nesting site selection, less attention has been given to hatching success, a key factor in reproductive outcomes. Indeed, site selection is only a part of the story, and reproductive success can only be fully understood by also investigating the process after it, i.e., hatching success, an overlooked aspect of the life cycle of sea turtles. In the present study hatching success is investigated for the first time in the case of loggerhead sea turtles in relation to multiple physical, chemical, biological, and anthropogenic parameters. Similar studies exist on leatherback (*Dermochelys coriacea*) and green (*Chelonia mydas*) sea turtles on Bioko Island, Equatorial Guinea [5].

In Italy, as in the broader Mediterranean region, the nesting season for the loggerhead sea turtle typically begins in early May and extends through August, while the hatching period generally occurs from late July to October. These patterns are well-documented in the literature, reflecting the seasonal reproductive cycle of loggerhead sea turtles, with variations influenced by local environmental conditions such as temperature and beach characteristics. Notably, the hatching period is highly variable, with differences in incubation duration driven by factors such as sand temperature, humidity, and local weather conditions [5]. Understanding how environmental and human conditions influence hatching success is critical to designing targeted conservation strategies to protect historical nesting beaches and suitable nesting sites. Indeed, coastal areas worldwide are facing a multitude of pressures resulting from the rising tourism industry, rapid urbanization, and the broader impacts of climate change [18]. In this context, the complexities of Italy’s coastlines exemplify global challenges, highlighting the delicate balance between human development and ecosystem preservation. The rich coastal biodiversity, diverse morphological features, and long history of human impact along Italian shores present a compelling case study for proactive international conservation efforts [19]. Moreover, Italy’s central location in the Mediterranean is crucial for understanding migratory shifts driven by climate change [20,21]. Therefore, this study aims to identify the key environmental and anthropogenic factors influencing the hatching success of loggerhead sea turtles along the Italian coastline, providing crucial insights for conservation and management strategies.

## Materials and methods

### Data collection

The dataset for this study includes nest records from 2019 to 2023, primarily sourced from Tartapedia (www.tartapedia.it), a real-time network aggregating data from public institutions and non-governmental organizations (NGOs) monitoring sea turtle nesting activities in Italy. To ensure accuracy, we cross-checked Tartapedia’s information with additional sources, including news articles and NGO reports, focusing on key metrics such as egg counts and hatching success. Each nest was geolocated, and egg-laying dates, additional data on nest translocation, egg counts, and meteorological impacts were obtained through direct contact with NGOs and social media posts. In some cases where detailed location data was missing, coordinates were estimated using QGIS [22] based on available pictures of the nest.

### Study area and nest selection

The Italian coastline, extending for approximately 8000 km, offers a diverse range of geographical and ecological features [23], capturing a large gradient of eco-morphological and anthropogenic conditions. Notably, around 41% of the coastline consists of sandy beaches, totaling about 3346 km suitable for nesting [24–26].

From the 1179 nests recorded on Tartapedia between 2019 and 2023 across 15 regions, 237 nests were selected for detailed analysis. These nests were chosen based on the availability of complete data, including the total number of laid eggs, hatching success, and precise geographical location, which are crucial for our statistical models.

### Predictors selection

The selection of predictors for the model aimed to integrate both natural and anthropogenic factors potentially influencing hatching success. A review of the literature was conducted to identify relevant variables [11,12,27,28], while additional predictors were included based on expert knowledge of the species’ ecology and potential conservation strategies. Key data sources, including the Copernicus program (https://www.copernicus.eu/en) and Google Earth Pro (https://earth.google.com), were used to obtain standardized, spatially and temporally detailed data across the study area and period. S1 Table lists the identified predictors, which are discussed below, along with their units of measurement and spatial resolution.

### Marine data

To assess the influence of marine environmental factors on sea turtle eggs during incubation, we used data from the Copernicus Marine Service [29–32]. The key variables analyzed included SST, nutrient concentrations (nitrate, ammonium, phosphate), primary production (PP), dissolved oxygen (DO), pH, dissolved inorganic carbon (DIC), alkalinity (ALK), surface partial pressure of CO_2_ (SPP), salinity (SAL), Wave Max. These indicators were averaged over the incubation period at the water surface (depth 0-1 m), using the pixel nearest to the nesting site. Wave Max was extracted as the maximum value during incubation to account for potential disturbances by extreme weather events. These variables are crucial for understanding how environmental changes impact nest conditions and egg survival. For instance, SST was discovered to be a good predictor of understanding the nest temperature [33], while nutrient levels reflect habitat quality [34] and, furthermore, ocean acidification can affect eggshell integrity [35]. Additionally, salinity [36] and wave disturbances [37] may directly impact nest viability.

### Terrestrial data

LST influences embryonic development and sex determination [38], these data were obtained from the Copernicus Sentinel-3 satellite via the Copernicus Land Service [39], thus providing a standardized dataset. The average daily temperature during the incubation period was calculated from the pixel nearest the nesting site, with the available spatial resolution of 5x5 km.

Nesting beaches were identified using Google Maps (https://www.google.it/maps) and classified by beach substrate type (BST) as sandy, mixed pebble-sandy, or pebble beaches based on comparisons through tourist photos available on Google Maps. Although substrate grain size is known to affect hatching success [40], standardized and comparable granulometric data are not available for all nesting sites in Italy. To ensure consistency and comparability between sites, we adopted a simplified approach by relying on surface observations and consequent classifications derived from photographic evidence. While this method is less precise than in-situ characterization, it provides a uniform and feasible framework for comparing a large number of beaches in the absence of local data.

Aerial images from Google Earth Pro were used to assess the beach’s morphology for the year of deposition. The beach width was measured from the shoreline to the highest point along a line drawn so that it was perpendicular to the nest. Then, the slope of the beach was determined using elevation profiles obtained from Google Earth Pro. Based on published data, the margin of error for these measurements is approximately ± 1-2 meters for width and ±  0.1° for slope, depending on the resolution of the satellite imagery [41]. To account for on-site variability, multiple measurements were taken, and the average values were used.

Human impact on each beach was assessed using two approaches: measuring human pressure within a 100 m radius of the nest [16] and calculating the distance to the nearest structures, considering the closest one as most relevant for disturbance. A 100 m radius was drawn on aerial images from Google Earth Pro to count buildings (N_House), road lanes (N_Roads), and beach umbrellas. Tourism pressure (TP) was categorized into three levels: low (<50 umbrellas), medium (50-100), and high (>100).

Beaches were classified using satellite imagery (Google Earth Pro) from the year of egg deposition as either “Anthropic” (with visible human-made structures like beach facilities, offshore structures, or breakwaters) or “natural” (without visible human intervention). This classification (Nat/Ant) aimed to assess the impact of anthropogenic factors on nesting sites. Additionally, distances from the nest to nearby human-made structures: buildings (F_House), roads (F_Roads) and beach facilities (F_Facilities), green areas beyond the beach (F_Green Zone; ≥  100m² of vegetation), and dunes (F_Dune) were measured as indicators of natural beach conditions.

### Missing data treatment

In case of missing laying (48 instances) or hatching dates (70 instances) due to unrecorded nests or gaps in grey literature, dates were estimated. For nests with complete data, the average incubation period was calculated by determining the difference between recorded hatching and laying dates. This average was then either subtracted from the hatching date or added to the laying date to estimate missing values. These estimations were used exclusively for gap-filling in statistical models, not for descriptive analyses.

### Correlation analysis and generalized linear models

To minimize model bias, correlation analysis among predictors was performed using Spearman’s rank correlation coefficient (rSpearman). Predictors with rSpearman >  0.70 were considered for exclusion, prioritizing those with clearer ecological or biological interpretability. A GLM [42] was developed to predict hatching success and identify key predictors. Different predictor combinations were tested and model selection was based on Akaike’s Information Criterion (AICc) [43] showing the most parsimonious model.

Given the zero-inflated data, multiple modeling strategies were adopted. The first GLM analyzed hatching presence or absence per each site using a binomial response (presence =  1, absence =  0). Model performance was assessed using the Area Under the Curve (AUC) of the Receiver Operating Characteristic (ROC), measuring sensitivity and specificity at various thresholds [43]. Due to zero hatching success in 31 nests, a second approach focused on nests with at least some hatching success, using a count matrix (successful and failed hatchings) to analyze influencing factors. This approach avoided assuming that the same factors causing complete failure also affected success in nests with at least one hatchling. A third GLM approach modeled both hatching and failure events per site using zero-inflated Poisson models [44] to address the excess zeros and improve predictor accuracy. The effectiveness of the second and third models was compared to a baseline model without predictors, assessing improvements in predictive accuracy.

All analyses were conducted in R-Studio, using libraries like readxl [45], MuMIn [46], PerformanceAnalytics [47], stats [48], ggplot2 [49], corrr [50], and pROC [51].

## Results

### Spatio-temporal patterns in nesting

The recorded nests over five years show a general increasing trend along the Italian coastlines, although with notable year-to-year fluctuations. For instance, in 2020, nest numbers increased by 161.7% compared to 2019 (from 94 to 246 nests). This was followed by a modest percentage increase of 4.1% in 2021 (256 nests). However, 2022 marked a temporary decline, with nest numbers dropping by 49.6% compared to 2021 (129 nests). In 2023, there was a significant increase of 252.7% compared to 2022, reaching 454 nests. These variations highlight the dynamic nature of nesting activity, influenced by both environmental and biological factors.

**Fig 1:**
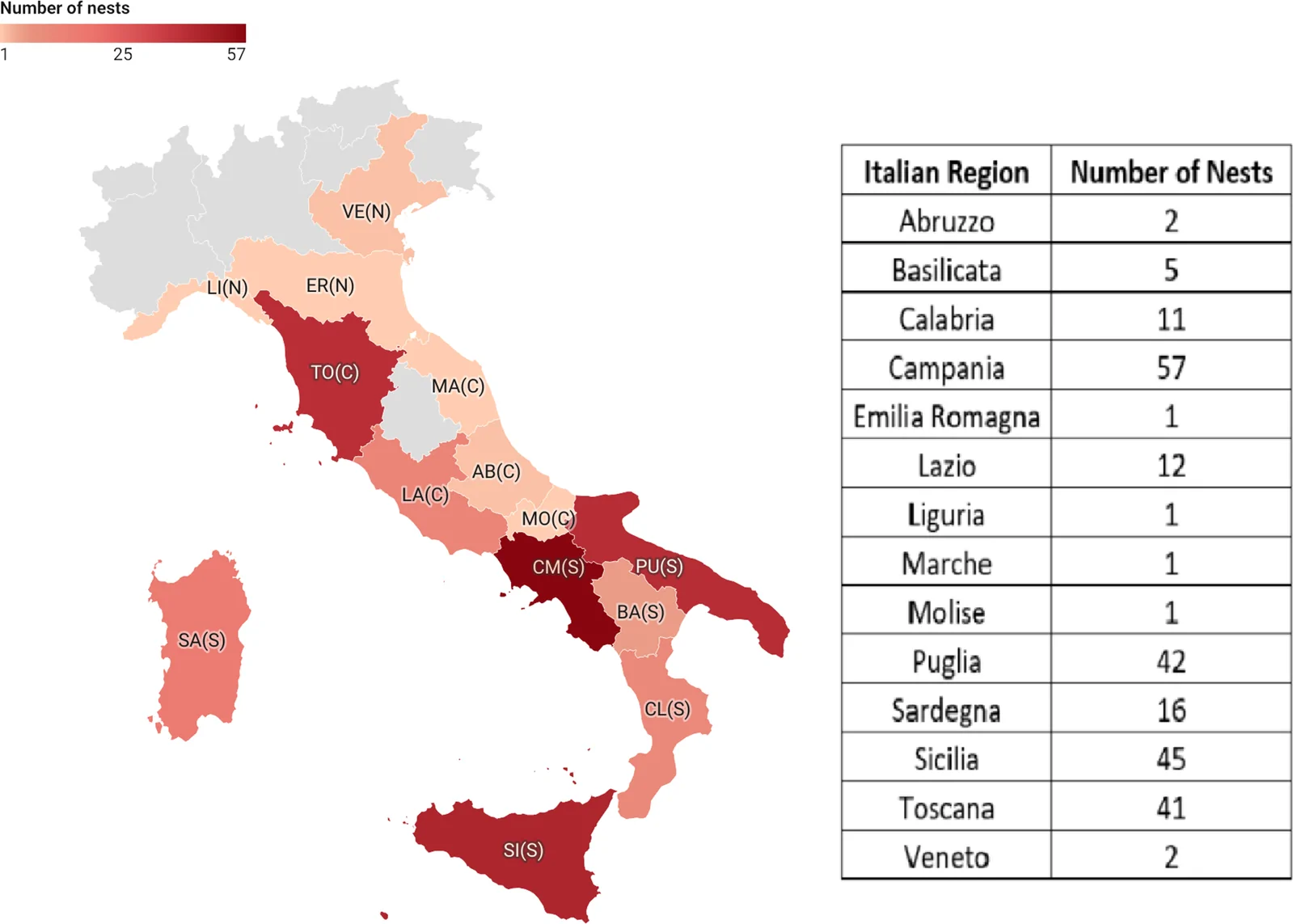
Regional distribution and geographical division of the analyzed nests across coastal regions of Italy over 2019-2023. (AB = Abruzzo; BA = Basilicata; CL = Calabria; CM = Campania; ER = Emilia-Romagna; LA = Lazio; LI = Liguria; MA = Marche; MO = Molise; PU = Puglia; SA = Sardegna; SI = Sicilia; TO = Toscana; VE = Veneto; N = North; C = Center; S =  South; n = 237) (*All utilized geographical data are under the Creative Commons Attribution License (CC BY 4.0). Software: QGIS 3.3. Note: Own elaboration*).

**Fig 2:**
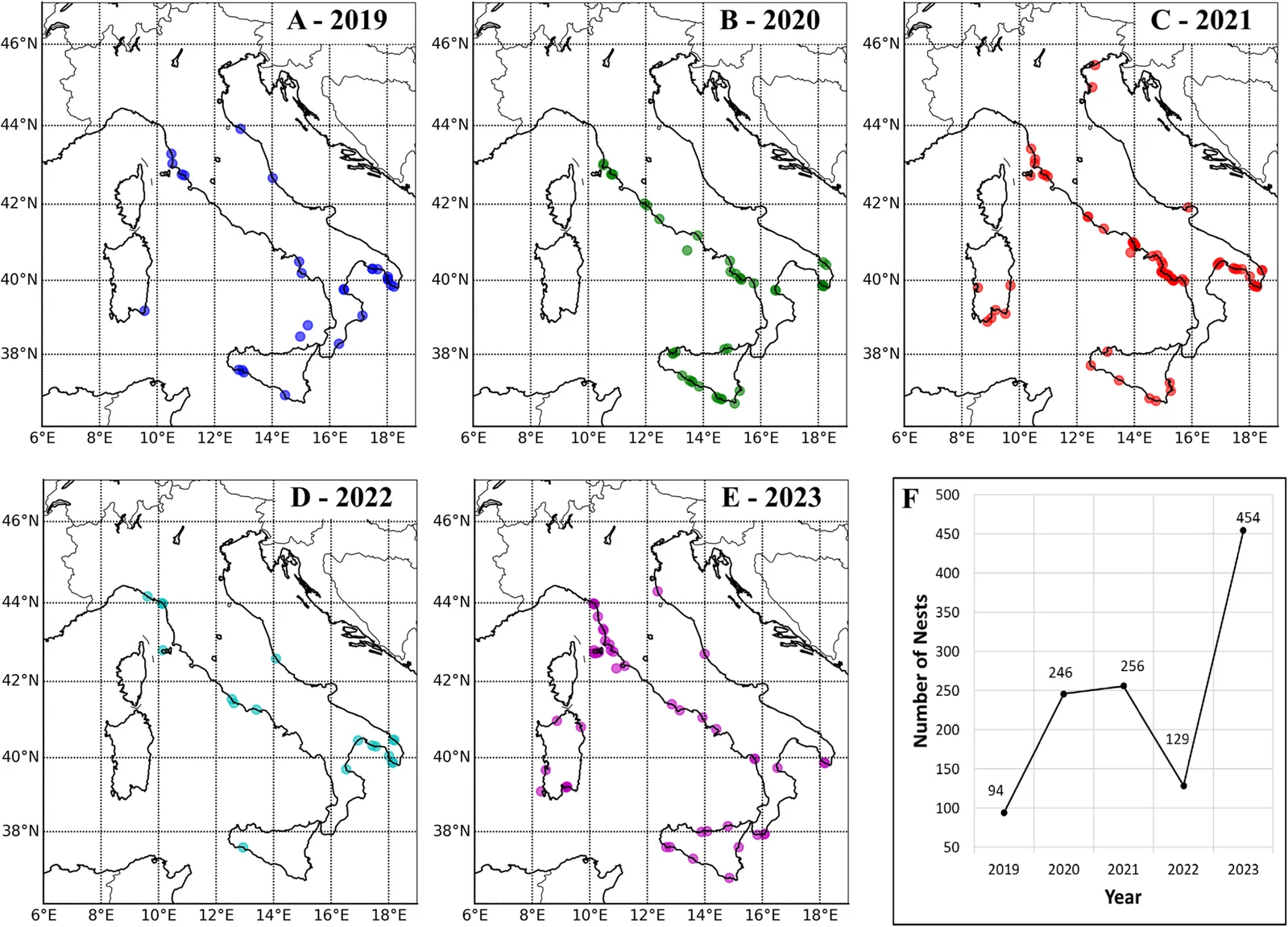
Distribution of nests along the Italian coasts divided by year (A:2019; B:2020; C:2021; D:2022; E:2023; F: Trend of registered nests along the Italian coast between 2019 and 2023; n = 237). (*All utilized geographical data are under the Creative Commons Attribution License (CC BY 4.0). Software: QGIS 3.3. Note: Own elaboration*).

The spatial analysis of the 237 selected nests (Figs 1, 2) shows that the highest records were concentrated in southern-central regions like Campania, Puglia, and Sicilia, while only a few nests were recorded in northern regions. Friuli Venezia-Giulia was the only coastal region with no historically recorded loggerhead sea turtle nests.

**Fig 3:**
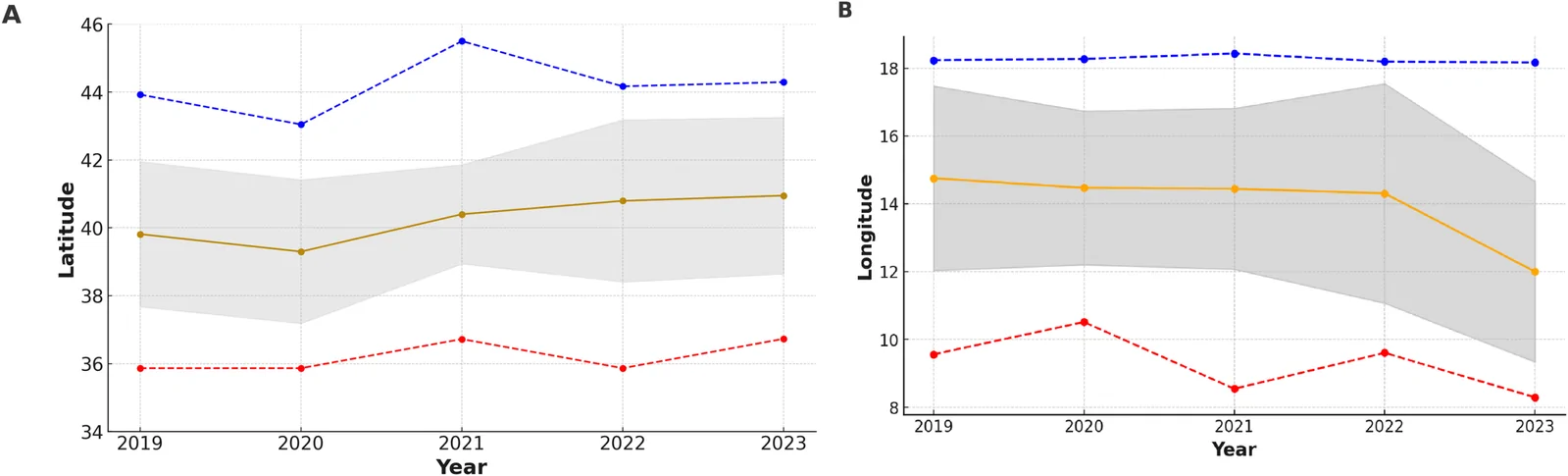
A: Trend of average latitude of the analyzed nest (n = 237) (blue line = max latitude, yellow line = mean latitude, red line = min latitude; grey area = standard deviation). **B: Trend of average longitude of the analyzed nest (n = 237)** (blue line = max longitude, yellow line = mean longitude, red line = min longitude; grey area = standard deviation).

We calculated the average latitude of recorded nests for each year (Fig 3A), which shows a positive trend over time (r_Pearson_ =  0.86, p =  0.058). Similarly, we calculated the average longitude of recorded nests, which shows a decreasing trend, approaching statistical significance (r_Pearson_ =  -0.79, p =  0.11), with a marked westward shift observed in 2023 (Fig 3B). These results indicate a potential expansion or shift in the nesting range both northward and westward.

**Fig 4:**
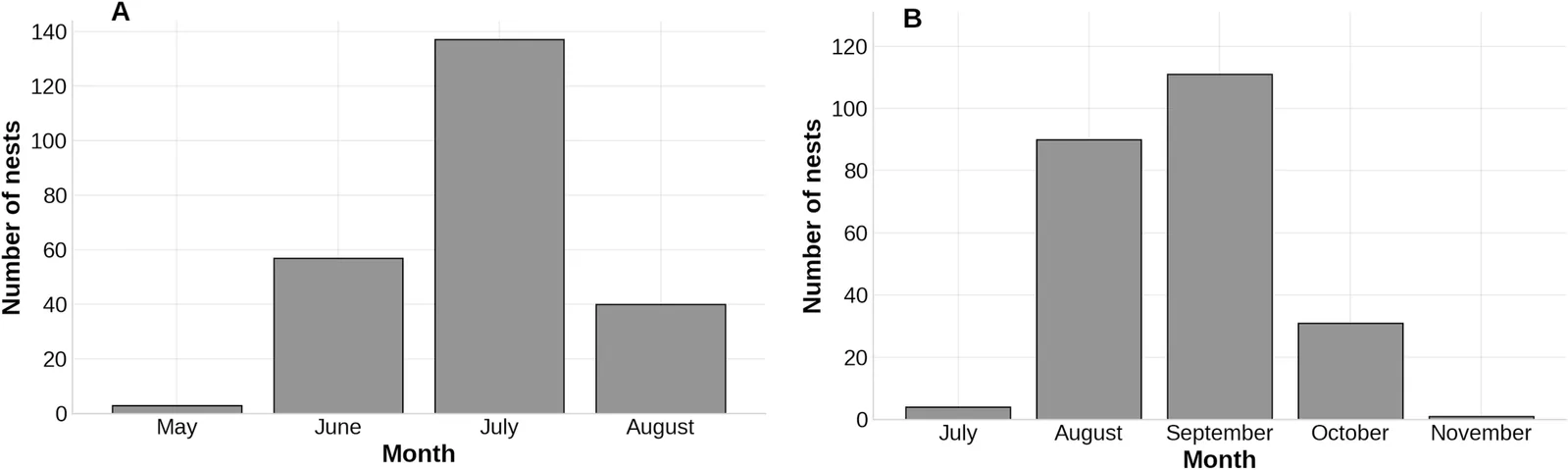
A: Nesting activity distribution (n = 237); B: Hatching activity distribution (n = 237).

The seasonal analysis of the 237 nests shows that nesting activity primarily occurs in late spring and summer, with most nests being laid in June (24.05%), July (57.80%), and August (16.88%), with only three depositions recorded at the end of May (1.27%) (Fig 4A). Hatching takes place from late summer to early autumn, mainly in August (37.97%), September (46.84%), and October (13.08%), with a minor peak in July (1.69%) and a single case in November (0.42%) (Fig 4B).

**Fig 5:**
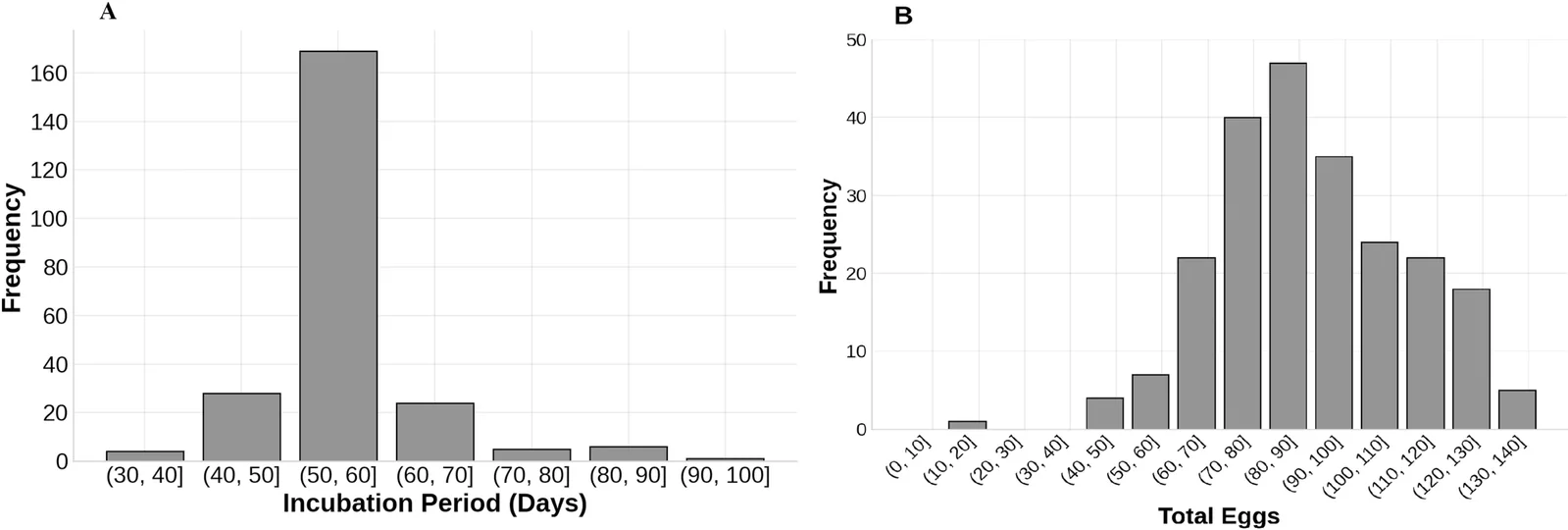
A: Incubation period grouped by 10 days (n = 237); B: Total eggs grouped by 10 (n = **237).**

The average incubation period was 56.4 ±  8.0 days, ranging from a minimum of 31 days to a maximum of 91 days. Notable cases included four nests with unusually short incubation periods from 30 to 40 days and seven nests with prolonged durations exceeding 80 days (Fig 5A). The mean clutch size was 90.5 eggs ±  20.56, with a minimum of 15 (an outlier, see Fig 5B) and a maximum of 134 eggs. In most nests (n = 225), the total number of eggs ranged from 43 to 134 (Fig 5B).

**Fig 6:**
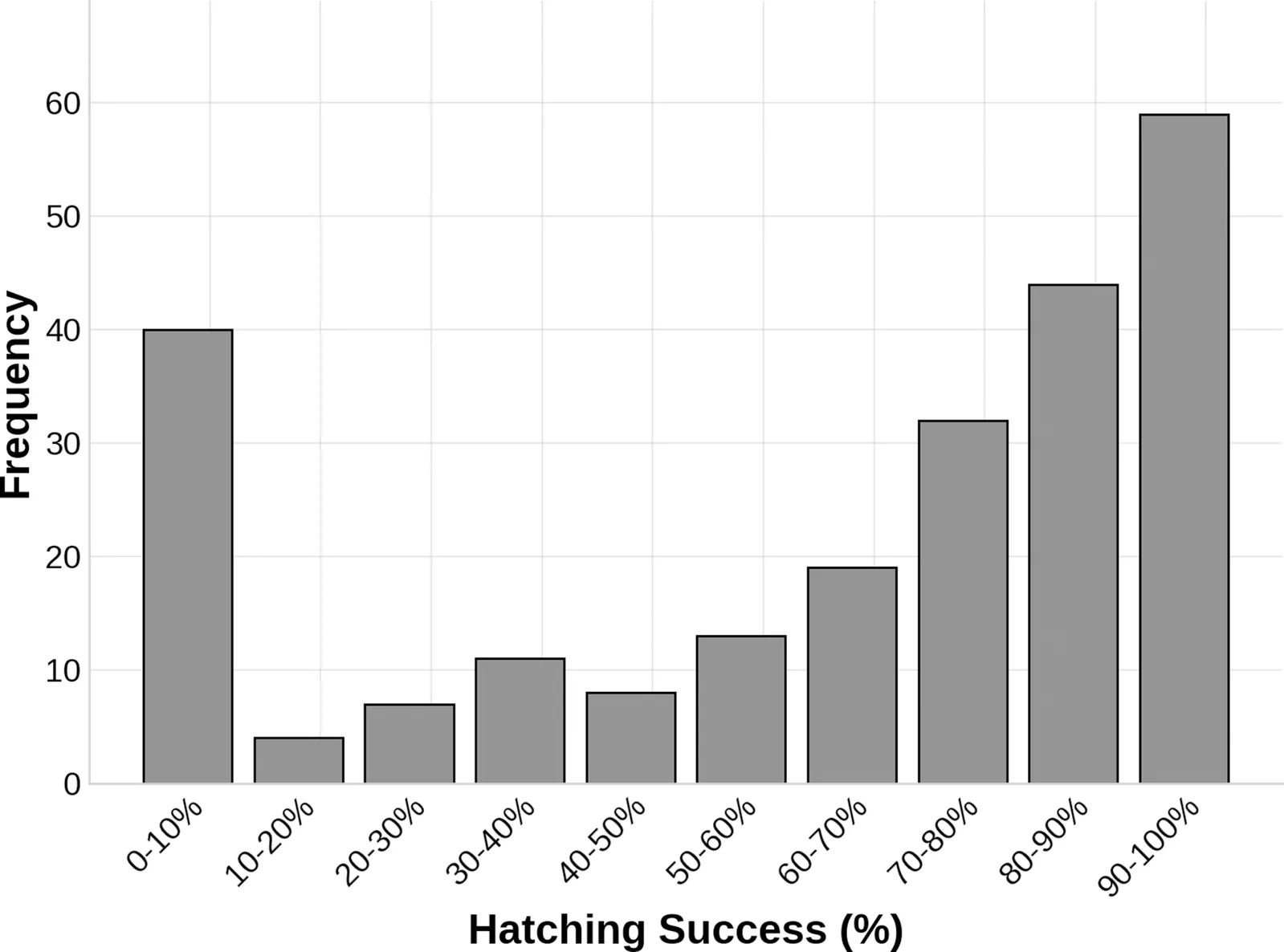
Hatching success along the Italian coast (nest success is grouped by 10%) (n = **237).**

The hatching success in the 237 analyzed nests (Fig 6) varied from lower than 10% (16.88%) to more than 60% (64.98%). There was a non-negligible number of nests (13.08%) where no egg hatched.

### Marine environmental indicators

Marine indicators (S2 Table) reveal diverse environmental conditions across the nesting sites. For instance, the average SST during the incubation period was 26.6°C, ranging from 23.37°C to 28.24°C for 236 nests (99.58%), with one early deposition in May in Sicilia showing a lower mean temperature of 22.57°C. Nitrate concentrations for 234 nests (98.73%) were below 5 mmol/m³, with three notable exceptions in the northern Adriatic Sea: two estuarine areas in the Veneto region near the mouths of the Po, Sile, and Piave rivers, where values reached 11.99 and 23.90 mmol/m³, and one nest in the Marche region near the mouth of the Foglia river with a concentration of 9.67 mmol/m³

### Beach morphology and coastal impacts

Regarding beach morphology (S3 Table), beach width ranged from 5.10 m to 67.4 m (S1A Fig), reflecting the varied characteristics of the Italian coastline. The beach slope (S1B Fig) ranged from 0% to 31%. In terms of Beach SubstrateType (BST), sandy beaches dominate, accounting for 87.76% of all nesting sites, followed by mixed beaches at 9.28% and pebble beaches at 2.95%.

The data highlight contrasting levels of natural conditions and human impacts across nesting sites, with significant spatial variability. Notably, 40.51% of the beaches lack dunes, with a consequent higher exposure to environmental and anthropogenic stressors. Where dunes are present, their distance (F_Dune) from the nests varies widely, ranging from just 3.96 m to 210.52 m, demonstrating a high degree of spatial heterogeneity in natural barriers.

All nests are located near green areas beyond the beach, with distances ranging from 8.86 m to 606.42 m from the nest to the closest vegetated zone, indicating that natural buffers against human activities and urban expansion are sometimes minimal. Human influence is evident, with 57.38% of nests within 100 m of at least one building, highlighting significant human intrusion. Additionally, 64.14% of nests are within 100 m of a road.

The proximity of nests to beach facilities is particularly noteworthy, with nearly half (49.37%) located less than 10 m away. The shortest distances to a building and a road are 12.2 m and 13.1 m, respectively, underscoring the potential closeness of nesting to human structures. In contrast, the most isolated nests are over a kilometer away from any infrastructure, offering some level of seclusion.

Tourism intensity varies greatly, with 39.66% of sites experiencing a high impact from tourist activities. This extends to the physical state of the beaches, where 39.66% remain in a natural condition, while 60.34% have been altered by human presence (Nat/Ant classification).

Conservation measures are frequently implemented, with 30.38% of nests having been translocated to mitigate environmental and human-related risks. Natural disturbances also pose challenges, with 5.06% of nests compromised by sea storms. Additionally, 17.72% of hatchlings emerged from previously undetected nests.

### Predictors

The final predictors after excluding redundant with the correlation matrix (S2 Fig) are displayed in Table 1. Descriptive statistical analyses were conducted exclusively on the selected predictors.

**Table 1:** Predictors selected for GLM models.

Selected predictors	Short Name	Type of Predictor
Nitrate concentration	NO_3_	Sea Characteristics
Dissolved Oxygen concentration	DO	Sea Characteristics
Maximum wave height	Wave Max	Sea Characteristics
Sea Surface Temperature	SST	Sea Characteristics
Beach Width	Beach Width	Beach Characteristics
Beach SubstrateType	BST	Beach Characteristics
Land surface temperature	LST	Beach Characteristics
Distance from the nest to the nearest building	F_House	Coastal Human Impacts
Natural (=0) or anthropized (=1) beach	Nat/Ant	Coastal Human Impacts
Number of roads within a 100 m radius from the nest	F_Roads	Coastal Human Impacts
Distance from the nest to the nearest dune	F_Dune	Coastal Human Impacts
Distance from the nest to the nearest green zone	F_Green Zone	Coastal Human Impacts

### Hatching success and nest variables

**Fig 7:**
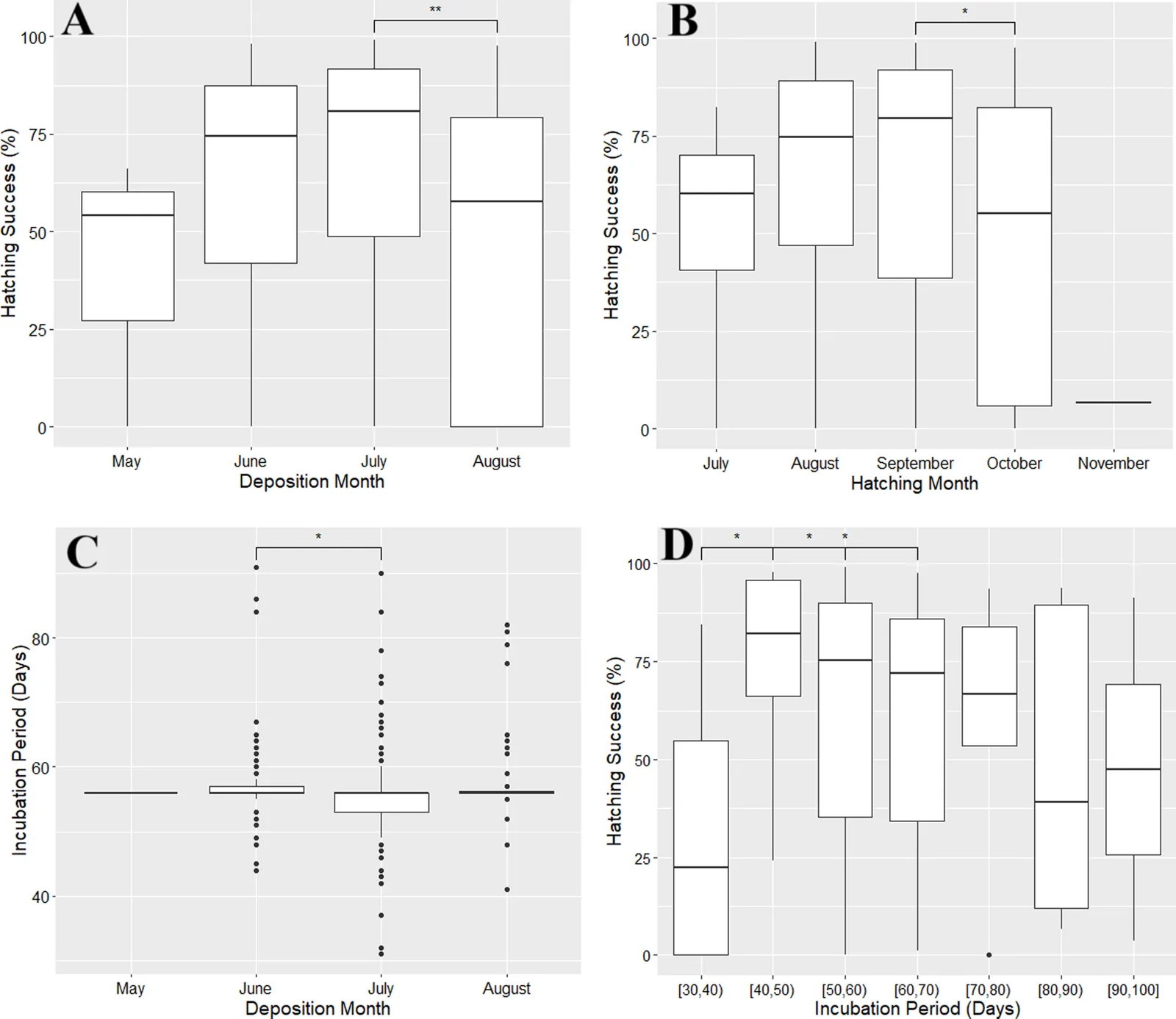
Bivariate relationships between A: Deposition Month and Hatching Success, B: Hatching Month and Hatching Success, C: Deposition Month and Incubation period (days), D: Incubation Period (in days) and Hatching Success (n = 237; * : p-value < 0.05; **: p-value < 0.01).

Significant statistical differences were observed when comparing hatching success rates across months (Figs 7A, 7B). Deposition in July showed higher hatching success than that in August (Dunn Test: -2.792, p = 0.003), indicating a decrease in success as deposition progresses into late summer. Similarly, hatching in September outperformed that in October in terms of hatching success (Dunn Test: -2.264, p =  0.011), pointing to a decline in success in autumn. The incubation period also varied significantly (Fig 7C); eggs deposited in June had longer incubation periods than those in July (Dunn Test: -1.971, p =  0.031). Further analysis of incubation duration (Fig 7D) revealed that shorter incubation periods (30-40 days) resulted in lower hatching success than longer ones (40-50 days; Dunn Test: -2.413, p =  0.004). Extending the incubation to 50-60 days and 60-70 days decreased hatching success rates compared to the 40-50 days period (Dunn Test: 2.188, p =  0.001 and 2.015 with p =  0.007, respectively).

By correlating hatching success with nest relocation, it was possible to observe a slight tendency for a difference in median values of success between translocation and no-translocation of the nests (Dunn Test =  1.371; p-value = 0.085), with relocated nests having a slightly higher hatching success (S3A Fig). The geographical division of Italy (IGD) in the north, center, and south regions did not show any significant difference in hatching success (S3B Fig). However, it should be noted that southern regions account for approximately 75.53% of the total number of analyzed nests.

### GLM models

The first modeling approach analyzed nests based on the presence or absence of any hatching [52] as the response variable. Several models performed similarly in terms of AICc (Table 2), and our selected models prioritize those with fewer parameters that avoid overfitting [53]. The best model (Model 549) included F_Dune, LST, and Nat/Ant as predictors. F_Dune and Nat/Ant were negatively correlated with hatching presence, while LST showed a positive correlation.

**Table 2:** Best GLM models ranked by AICc.

Model n.	F_House	F_Dune	BST	LST	Nat/Art	Wave Max	AICc	ΔAICc
549		-0.001		0.188	-0.822		177.320	0.000
563	0.003		0.645	0.187	-0.815		177.356	0.036
2579	0.002		0.729		-1.024	-0.343	177.464	0.145
551	0.002	-0.001		0.181	-0.792		177.535	0.216
2565		-0.001			-0.984	-0.329	177.629	0.309
805		-0.001		0.203	-0.864	-0.327	178.68	1.360
2581		-0.001	0.5536		-1.081		178.931	1.611
2611	0.002		0.656	0.134	-0.882	-0.240	179.263	1.943

Another notable model, Model 2565, included F_Dune, Nat/Ant, and Wave Max, with Wave Max negatively influencing hatching success during the incubation period. Similarly, Model 2581 had a comparable ΔAICc ( < 2) and retained the negative effects of F_Dune and Nat/Ant while adding BST as a predictor, where sandy beaches were positively associated with hatching success.

The AIC values for Models 549, 2565, and 2581 were 0.742, 0.740, and 0.733, respectively, indicating reasonable predictive ability in distinguishing between hatching and non-hatching nests.

In the second GLM model approach (Table 3), which analyzed nests with hatching success >  0%, three models showed similar AICc values (ΔAICc <  2): Models 3968, 3712, and 4096. All three models included the same predictors: Beach Width (positively correlated with hatching success), F_Dune (negatively correlated with hatching success), BST (sandy beaches were associated with higher hatching success), LST (positively correlated with hatching success), Nat/Ant (lower hatching success on anthropized beaches), and Wave Max (negatively correlated with hatching success).

**Table 3:** Best GLM models ranked by AICc.

Model n.	Beach Width	F_Dune	BST	LST	Nat/Ant	Wave Max	AICc	ΔAICc
3968	0.013	-0.001	0.304	0.074	-0.100	-0.110	6474.898	0.000
3712	0.013	-0.001	0.308	0.075	-0.110	-0.114	6476.516	1.618
4096	0.013	-0.001	0.306	0.072	-0.103	-0.109	6476.695	1.796
3840	0.013	-0.001	0.311		0.113	-0.112	6477.695	2.797
3456	0.013	-0.001	0.281	0.081		-0.113	6479.426	4.528

The significant reduction in residual deviance indicates the relevance of these predictors for predicting hatching success. For Model 3968, deviance decreased from 630.8 to 110.2 (p <  0.001); for Model 3712, from 630.8 to 105.2 (p <  0.001); and for Model 4096, from 630.8 to 143.5 (p <  0.001).

In the third GLM model approach (Table 4), nests were analyzed as a matrix of hatched and non-hatched eggs. Models 3966 and 4093 had the best AICc values, both including the same predictors: Beach Width (positively correlated with hatching success), F_House (positively correlated with hatching success), F_Green Zone (negatively correlated with hatching success), BST (sandy beaches were associated with higher hatching success), LST (positively correlated with success), Nat/Ant (lower hatching success on anthropized beaches), and Wave Max (negatively correlated with hatching success).

**Table 4:** Best GLM models ranked by AICc.

Model n.	Beach Width	F_House	F_Green Zone	BST	LST	Nat/Ant	Wave Max	AICc	ΔAICc
3966	0.009	0.001	-0.002	0.477	0.095	-0.456	-0.043	10492.221	0.000
4093	0.009	0.001	-0.002	0.478	0.094	-0.458	-0.042	10493.747	1.526
1920	0.009	0.001	-0.002	0.472	0.087	-0.459		10496.681	4.459
2048	0.009	0.001	-0.002	0.474	0.086	-0.461		10497.707	5.486
3964	0.008	0.001	-0.002	0.508	0.098	-0.445	-0.035	10504.009	11.787

The effectiveness of these models was tested against a model without predictors, showing a significant improvement in predictive ability. For Model 3966, deviance was reduced from 630.8 to 327.58 (p <  0.001), while for Model 4093, deviance decreased from 630.8 to 312.6 (p <  0.001).

## Discussion

The recent increase in the number of loggerhead sea turtle nests along the Italian coasts can be attributed to a combination of natural and anthropogenic factors. Climate change is influencing the reproductive behavior of this species, facilitating an expansion of its nesting range toward the north and west of the Mediterranean Sea [7]. Rising sea temperatures, in particular, are creating more favorable conditions for nesting in areas previously considered marginal or unsuitable.

On the other hand, increased conservation efforts and enhanced monitoring by local organizations, citizen science initiatives, and governmental bodies have likely contributed to identifying and protecting a larger number of nests. These efforts include systematic beach patrols, awareness campaigns, and the implementation of protective measures at nesting sites. This combined approach not only ensures the preservation of nests but also enhances data collection, potentially explaining the observed increase in nesting records [54]. In the absence of an official, standardized Italian database for sea turtle nesting activities, the collaboratively managed platform (Tartapedia), from which data were obtained, represents an example of a successful citizen science approach, increasingly used in marine research [55]. However, despite the database’s wide scope enabling valuable large-scale and long-term analysis, the data quality may vary and contain biases; we tackled this issue by comprehensive data cross-checks and supplementary information from official sources and scientific publications, revealing that data quality is generally sound. The use of citizen science data coupled with online resources allowed us to collect large datasets across all relevant Italian regions, facilitating biogeographic analysis. Utilizing modern remote and standardized data store tools like Copernicus, we could evaluate multiple factors affecting hatching success, incorporating marine environment elements systematically for the first time and making it a pioneering effort in the field. Finally, the GLM approach allowed us to deal with non-normally distributed data and multiple variables, while assessing model performance with information theory to select the most parsimonious model.

When constructing the models, we filled in some missing data based on the average incubation period, consistently observed to be around 56 days, similar to other regional studies [56]. We provide new and robust data on the seasonality of nests and the duration of incubation on an Italian scale, significantly contributing to the understanding of these essential aspects of loggerhead sea turtle biology. Our study provided new insights into the incubation duration and the timing of laying and hatching phases. Nesting occurs mainly from June to August, with hatchings from August to October. Hatching success varied by month, with higher success when laying eggs in July compared to August, and with hatching in September outperforming October. Eggs laid in June had longer incubation periods than those laid in July. Shorter incubation periods (30-40 days) had lower hatching success than longer ones (40-50 days), and extending incubation to 50-70 days also decreased success. The success of hatching varies over time, likely due to the seasonal fluctuations in both human and natural pressures that impact nest success (such as sea storms, temperature changes, and tourist presence). The optimal hatching success is achieved within specific time frames, particularly during periods with favorable environmental conditions and minimal disturbances, highlighting the importance of the timing and duration of the incubation period. We observed significant variation in hatching success, with some nests exceeding 60% success and others failing due to factors like infectious diseases [8] or environmental conditions such as flooding and extreme weather, together with several other human and natural factors highlighted by our modeling approach. This finding emphasizes the need for monitoring and protective measures at nesting sites.

The consistency of predictor effects across our three modeling approaches reassured us of the robustness of the identified correlations. Hatching success was negatively related to the distance from dunes, green areas, the artificial state of the beach, and maximum wave height during incubation. Conversely, it was positively correlated with distance from buildings and beach width, supporting our hypothesis that less disturbed, natural beaches favor sea turtle hatching [57]. Additionally, land surface temperature and sandy beaches positively impacted hatching success, indicating that optimal thermal conditions and beach substrate are crucial for preventing diseases and promoting healthy embryonic development. The negative correlation with maximum wave height underscores the importance of sea storms and the protective role of dunes against them, as well as the significance of human interventions, such as nest protection measures, to mitigate the impact of sea storms.

Many of the detected factors affecting hatching success are under human responsibility. High levels of beach anthropization significantly decreased hatching success, with nearly half of the studied beaches hosting tourist facilities very close to nests. This overlap between tourism and nesting sites poses conservation challenges, so narrower beaches increase risks such as flooding or excessive tourist density, impeding turtle nesting success [58]. From a conservation perspective, it seems that maintaining broad, natural beaches with dunes and green areas is essential for sea turtle nesting. Our findings emphasize the importance of physical and thermal terrestrial characteristics over marine water quality in predicting hatching success, an indication of the strong dependence of the sensitive hatching phase on local beach conditions which has important implications for conservation actions that may successfully be carried out on site. These results show the potential of remote sensing and citizen science-based approaches to identify and monitor key predictors of nesting success, aligning with existing literature. We conclude that coastal urban planning should integrate ecological considerations to minimize anthropogenic impacts. While our study provides valuable insights, some limitations should be acknowledged. Missing data required estimations, particularly for nest deposition and hatching dates, which may introduce some uncertainty. Additionally, beach width and vegetation cover assessments relied on remote sensing, which may not fully capture local variations. Lastly, our model does not explicitly consider some localized disturbances, such as predator activity or unrecorded human interventions. Future studies could refine these aspects with in-situ monitoring and higher-resolution data.

This study identifies the main environmental and anthropogenic factors influencing the hatching success of loggerhead sea turtle nests along the Italian coastline, integrating previous research with new predictors based on remote sensing and citizen science. The findings provide crucial insights for enhancing beach management and nest protection, including potential relocation, involving key stakeholders in territorial planning, beach nourishment, and habitat conservation (e.g., dunes). Protective measures are especially urgent given the increasing human pressure along the Italian coasts, which could cause significant population-level damage [7,59,60].

Ideal nesting beaches for loggerhead sea turtles should have low human impact [57], proximity to dunes and vegetation, and sand that promotes optimal oxygenation and humidity, reducing the risk of fungal and bacterial growth [61]. Climate change impacts, such as rising sea levels and increasing temperatures [62], may alter nesting sites and affect the sex ratio, leading to potential range shifts and changes in deposition areas.

Our study suggests that most predictors of hatching success are within human control, emphasizing that appropriate management can create more favorable habitats. Although focused on Italy, our findings could be adapted to similar ecological zones globally, particularly in the Mediterranean Sea. Future research should include additional factors to enhance understanding of conservation strategies. Strengthening monitoring and data collection efforts remains essential for developing effective conservation measures to ensure the long-term survival of vulnerable species in the face of ongoing environmental challenges.

## Supporting information

S1 Table Summary of marine environmental characteristics along the Italian coast, showing the mean (with standard deviation), minimum, and maximum values assessed at sea close to each of all nests (237). All indicators represent averages over the incubation period of the corresponding nest, except for wave height which is the maximum over the incubation period.
(XLSX)

S2 Table Summary of beach morphology and coastal human impacts along the Italian coast, showing the mean, minimum, and maximum values measured across all nests (237). Land surface temperature is the average over the incubation period of the corresponding nest.
(XLSX)

S3 Table List of potential predictors. (XLSX)

S1 Fig A: Beach width frequency and B: Beach slope frequency for the sites of the analyzed nests (n = 237). (TIF)

S2 Fig Correlation matrix with all predictors identified (n = 237): (Transl=Nest Translocation; Succ = Nest Success; Width=Beach Width; Slope = Beach Slope; F_Facilities =  Distance from the nest to the nearest beach facilities; F_Roads =  Distance from the nest to the nearest roads; F_House =  Distance from the nest to the nearest building; ‘F_Green Zone = Distance from the nest to the nearest beach dunal zone; N_House =  Number of buildings in a radius of 100 m from the nest; N_Road =  Number of roads in a radius of 100 m from the nest; TP =  Tourism Pressure; Nat/Ant =  Natural (=0) or anthropized (=1) beach; BST =  Beach Substrate Type; NH4 = Ammonium Concentration; DO =  Dissolved Oxygen; NPPV =  Net Primary Production Value; DIC =  Dissolved Inorganic Carbon Concentration; ALK =  Alkalinity; pH; PO4 =  Phosphate Concentration NO3 =  Nitrate Concentration; SAL =  Salinity; SST =  Sea surface Temperature; LST =  Land Surface Temperature; IGD =  Italian Geographical Division, Wave Max =  Maximum wave height. (TIF)

S3 Fig Bivariate relationships result between A: Nest translocation and Hatching success, B: Italian geographical division and Hatching Success (n = 237). (TIF)
