# Expression of Concern: Modeling the impacts of natural and human factors on the hatching success of the loggerhead sea turtle *Caretta caretta* along the coasts of Italy

**DOI:** 10.1371/journal.pone.0361847

**Published:** 2026-11-03

**Authors:** The PLOS One Editors

## Notice of Republication

This article was republished on October 15, 2026, to address an issue identified post-publication. Please download this article again to view the correct version.

After this article [1] was published, concerns were raised about the validity of the reported data sources and the underlying dataset. As reported in [1], the dataset for the study was compiled from information reported on Tartapedia, which collects and shares approximate municipality-level nesting data provided by public institutions and non-governmental organizations. Data from weekly reports and news reports shared by Tartapedia during nesting season were cross-checked against additional public sources of information in the gray literature. Concerns were raised about the following:

- Potential inaccuracies in the reproductive data underlying Figs 1-6, which were gathered from secondary sources; the reported minimum incubation period of 31 days may be considered abnormally low for this species.
- Potential inaccuracies in the estimated geolocation data of nesting sites based on images used in news reports which may be unrelated representative images, affecting the reliability of the human impact data and marine and terrestrial environmental data related to the nest locations.
- The complete list of news reports used to generate the underlying dataset and the individual-level quantitative data underlying the reported results have not been publicly shared with the article [1].

PLOS consulted an independent expert who advised that reliance on data from secondary sources such as social media and press releases may affect the reliability of the results, but they noted that the overall results were expected.

The corresponding author stated that nesting and hatching data were obtained primarily from Tartapedia [2], including annual reports and the news section. Information from Tartapedia was cross-referenced with other public sources. Only reports which included complete information on clutch size, hatching success, and precise geographic location were used. The authors stated that their gap-filling procedure to account for missing dates and location was transparently reported in [1], stating that the estimates were only used for statistical modeling, not for descriptive analyses, and that the resulting values are fully consistent with those reported in the literature.

Regarding the low minimum incubation period, the corresponding author agreed this value is lower than expected, but they chose not to remove the few outliers so as not to introduce biases into the data. They indicated that these outliers could have resulted from minor imprecision from citizen reports of nest sightings. PLOS notes that the low minimum incubation period was acknowledged in the Results section of [1].

In response to the concerns about the accuracy of the geolocation data, the corresponding author stated that these data were not based solely on photographs in news reports but in most cases detailed location information was obtained directly from the original source. When supplementary information from newspaper articles or social media posts was used, photographs were examined together with information from the text, with careful consideration as to whether the image depicted the specific nesting event or if it was a representative image, which was also normally made clear by the media outlet. The corresponding author acknowledged that there may be uncertainty in the geolocation data for some nesting records but stated that any such uncertainty is unlikely to affect the study results and conclusions because environmental and anthropogenic variables were extracted at a spatial resolution of approximately 5 × 5 km, which is substantially larger than any plausible georeferencing error associated with individual nesting sites.

The corresponding author provided links to the Tartapedia annual reports that were used as the primary source of data for this study (S1 File); however, the accessibility of the report links appears to be unreliable at the time of preparation of this notice. The corresponding author also provides a list of available additional original sources used in the analysis and the individual-level quantitative data underlying all figures in S2 File. They have clarified that for 178 of 237 nesting events, one or more original online sources remain available, but 59 of the original webpages are no longer accessible because they have been removed or archived by the hosting websites.

In the second, fifth, and sixth paragraphs of the Discussion in [1], the analysis of data primarily derived from Tartapedia [2] is referred to as a citizen science approach. With this notice, the corresponding author clarifies that the information collected through Tartapedia relied substantially on contributions from a broad network of stakeholders involved in sea turtle conservation, including citizens [3], adding that citizen science is a flexible concept as noted by the European Citizen Science Association (ECSA) [4]. They stated that the text was not intended to imply that Tartapedia formally fulfils every ECSA principle, but to recognize the role of public participation in generating these data.

PLOS considers that the key finding of the article is to present a statistical model and this model may still provide value to readers. The article includes discussion on the data quality and biases; however, the concerns around the reliability of the underlying data, and how this may affect the validity of the statistical model, could not be fully resolved. The *PLOS One* Editors issue this Expression of Concern to provide additional information about the methodology and data sources and to make readers aware of the above limitations when interpreting the specific results reported in this article.

## Supporting information

S1 File Tartapedia annual report links. (DOCX)

S2 File Data sources and quantitative data underlying all figures. Available data sources include weekly reports, news articles, institutional webpages, and other web-based reports used during data collection.
(XLSX)
