# Supplementary figures and images for: Modeling the impacts of natural and human factors on the hatching success of the loggerhead sea turtle Caretta caretta along the coasts of Italy
Source: PLoS One. 2025 Apr 9;20(4):e0320733. doi: 10.1371/journal.pone.0320733 (PMC11981214; doi:10.1371/journal.pone.0320733)

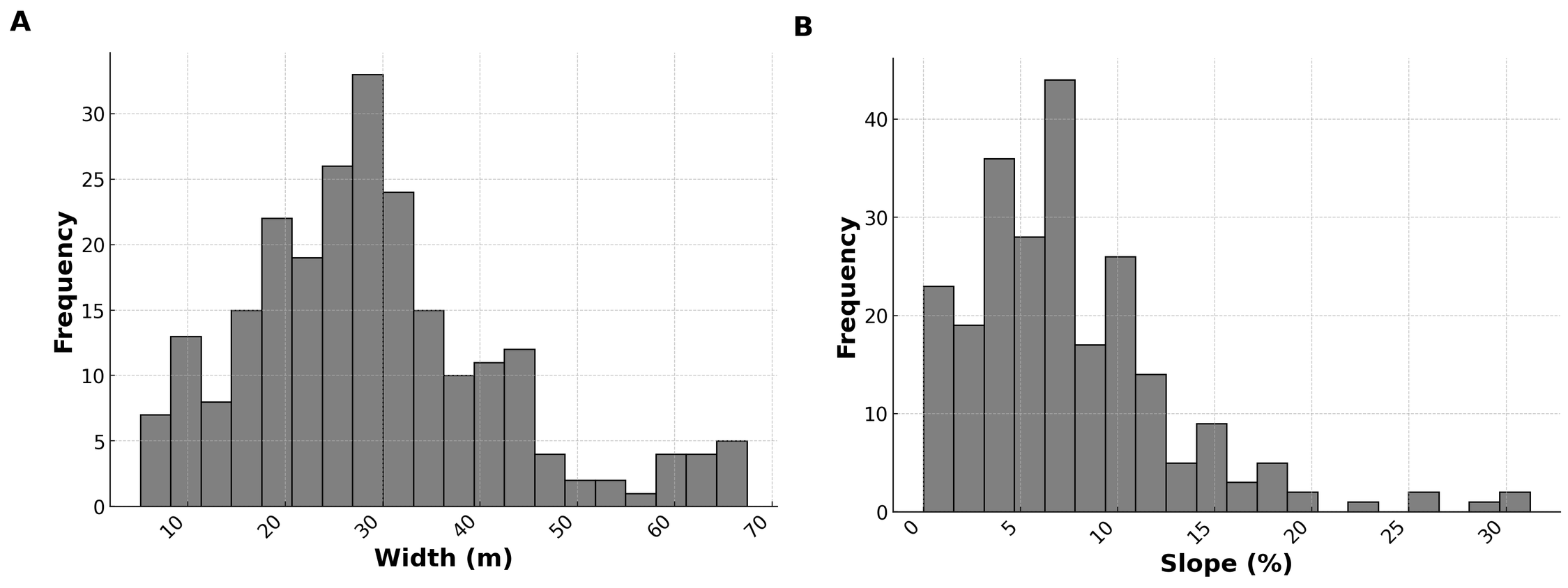

Supplement: S1 Fig — (TIF) [file pone.0320733.s004.tif]

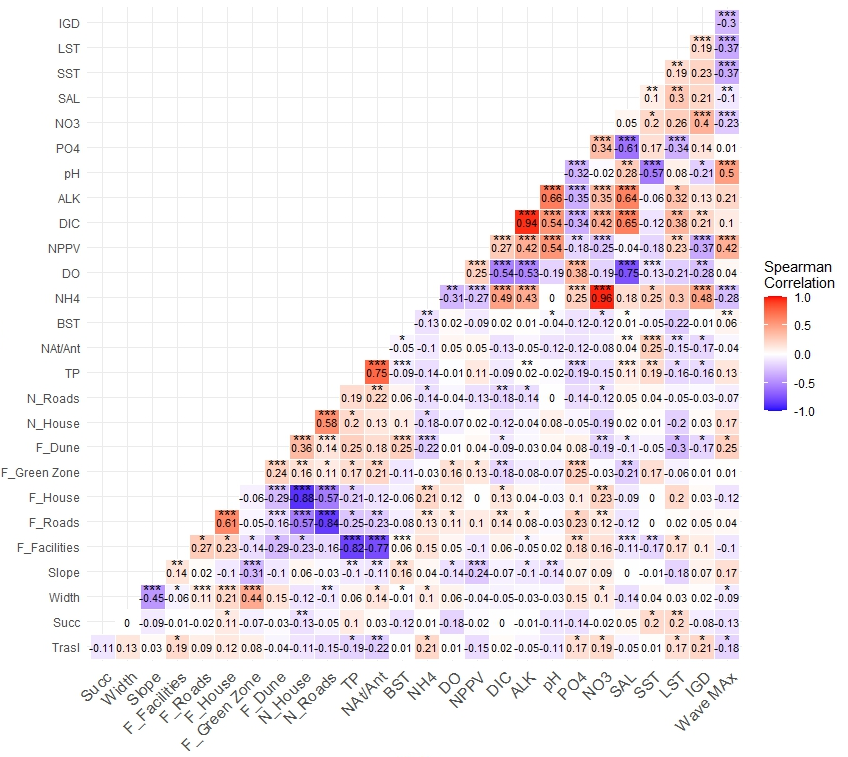

Supplement: S2 Fig — (TIF) [file pone.0320733.s005.tif]

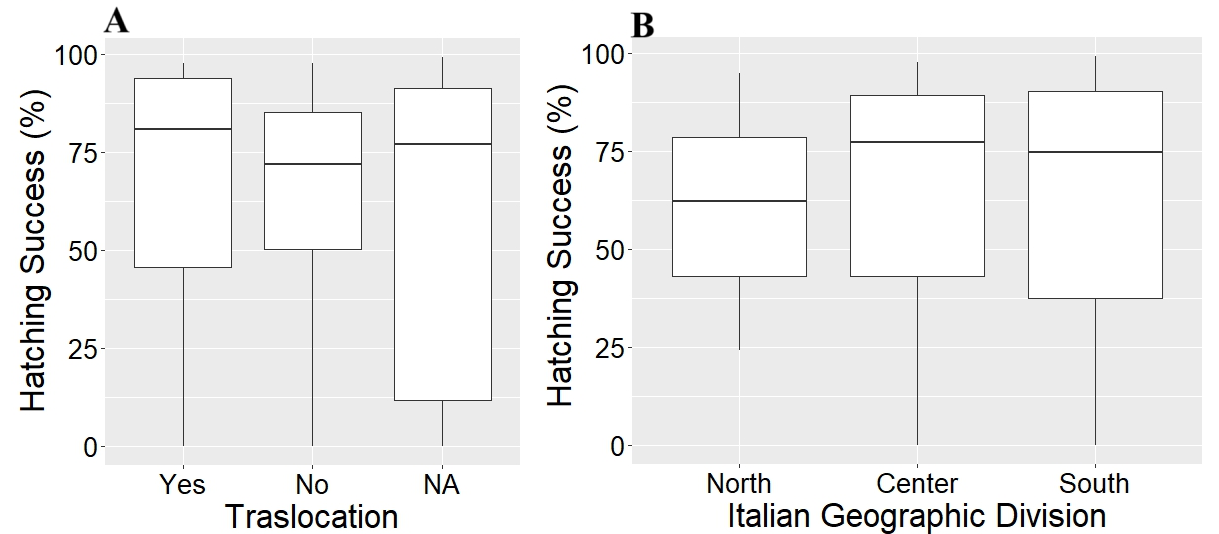

Supplement: S3 Fig — (TIF) [file pone.0320733.s006.tif]
