# Supplementary material for: Expression of Concern: Modeling the impacts of natural and human factors on the hatching success of the loggerhead sea turtle Caretta caretta along the coasts of Italy
Source: PLoS One. 2026 Nov 3;21(11):e0361847. doi: 10.1371/journal.pone.0361847 (PMC13730360; doi:10.1371/journal.pone.0361847)
Supplement: Supplementary file 1 [file pone.0361847.s001.docx]

**S1 File: Links to Tartapedia annual reports**

<https://tartapedia.it/decimo-aggiornamento-deposizioni-2019-di-caretta-caretta/>

<https://tartapedia.it/deposizioni-2020-di-caretta-caretta-in-italia-19esimo-aggiornamento/>

<https://tartapedia.it/nidificazioni-2021-di-caretta-caretta-in-italia-quindicesimo-aggiornamento/>

<https://tartapedia.it/nidi-di-caretta-caretta-in-italia-17esimo-aggiornamento-del-2022/>

<https://tartapedia.it/nidificazioni-di-tartaruga-marina-in-italia-19esimo-aggiornamento-del-2023/>
